# Quantitative Distribution Characterization and Correlation Study of Composition, Structure and Hardness of Rim Region in Railway Wheel

**DOI:** 10.3390/ma15144762

**Published:** 2022-07-07

**Authors:** Dongling Li, Haizhou Wang, Xuejing Shen, Shuangping Lin, Haozhou Feng, Ya Peng, Fan Jiang, Xuefan Zhou

**Affiliations:** 1 Beijing Advanced Innovation Center for Materials Genome Engineering, Beijing 100081, China; lidongling@ncschina.com (D.L.); shenxuejing@ncschina.com (X.S.); 2 Central Iron & Steel Research Institute, Beijing 100081, China; fenghaozhou@mails.ucas.ac.cn (H.F.); pengya18@mails.ucas.ac.cn (Y.P.); jiangfan20@mails.ucas.ac.cn (F.J.); zhouxf1217@mails.jlu.edu.cn (X.Z.); 3 Beijing Key Laboratory of Metallic Materials Characterization, Beijing 100081, China; linshuangping@ncschina.com; 4 NCS Testing Technology Co., Ltd., Beijing 100081, China

**Keywords:** tread, quantitative distribution, ferrite, inclusion, hardness, rim-chilling process

## Abstract

The railway wheel is the key component of high-speed railway train. To assure the safety in service, higher requirements are put forward in this study for the composition, microstructure uniformity, and comprehensive properties of wheel materials. In this paper, the high throughput quantitative distribution characterization methods of composition, microstructure, inclusions and Vickers hardness of high-speed railway wheel materials based on the spark source original position analysis technique, high throughput scanning electron microscope (SEM) combined with image batch processing technology, and automatic two-dimensional quantitative distribution analysis technique of inclusions and micro hardness have been studied. The distribution trend of the content of nine elements, size and quantity of sulfides and oxides, ferrite area fraction, and Vickers hardness from the wheel tread surface to the radial depth of about 50 mm below the surface has been discussed. The influence of inclusions distribution on the element segregation and the effect of rim-chilling process with different water spraying angle on the distribution of microstructure and micro hardness have been investigated. It was found that unsynchronized cooling on both sides of the rim altered the phase behavior of ferrite and pearlite and obvious inhomogeneity distribution of ferrite appeared, which led to the asymmetrical Vickers hardness in areas near or away from the flange. Based on the quantitative characterization of area fraction and micro hardness on the same location of wheel rim, a statistical mapping relationship between ferrite area fraction and Vickers hardness was established.

## 1. Introduction

The railway wheel is the key component of high-speed railway train and has been used in a demanding environment with high normal contact forces and significant tangential forces. The resulting stresses often exceed the yield stress of the as-manufactured wheel material, leading to plastic flow, wear, and fatigue damage [1,2,3]. Therefore, higher requirements have been put forward for the composition, microstructure and comprehensive properties of wheel materials to assure the safety in service [4]. Non-metallic inclusions are often the main cause of wheel fracture or fatigue failure [5,6,7,8] and the stress concentration around the inclusion easily leads to the formation of micro cracks and holes with the results of the deformation of the matrix and the formation and development of large cracks. Steels used for high-speed railway wheel are often designed with ultra-low oxygen content (≤15 ppm) and moderate sulfur content (≤150 ppm) to diminish the influence of non-metallic inclusions on catastrophic failures. The microstructure of medium carbon wheel steel is mostly composed of lamellar pearlite and a small amount of proeutectoid ferrite [9]. The composition and microstructure of high-speed wheels have great influence on the hardness and impact toughness of high-speed wheels. The addition of elements C, Si, Mn and other microalloying elements plays an important role in the final optimization of microstructure and properties [10,11,12,13,14,15]. The element segregation or non-uniform microstructure distribution in the key area from the wheel tread surface to the radial depth of about 50 mm will seriously affect the quality and performance of railway wheel [16,17,18,19]. Therefore, it is necessary to comprehensively characterize the composition, microstructure and properties distribution of the key parts of the wheel rim, and to explore the effect of heat treatment process on the distribution of microstructure and micro hardness. It will be helpful for the parameter optimization of heat treatment process and improve the performance of wheel rim.

At present, there are few studies on the composition distribution of elements, the quantitative distribution of inclusions and microstructure in the key area of railway wheel rim. The characterization of segregation degree for the full surface of large size high-speed train wheel was carried out based on the original position analytical statistical distribution technique [20]. But the elemental quantitative distribution on the key area of wheel rim has not been discussed and the influence of microstructure on the elemental distribution has not been investigated. In fact, the different microstructure such as ferrite, pearlite and bainite and their volume fraction distribution have great influence on fatigue crack resistance in the wheel steel. It was found that fatigue micro-cracks mostly initiated close to interface regions between soft and hard phases. The bainitic phase could obviously retard fatigue crack propagation of steel, whereas the ferrite contrarily exhibited deteriorated crack resistance [21]. Tian et al. [22] investigated the hardness on rim sides and found that the quenching process parameters had great influence on the hardness distribution. But the relationship between the microstructure homogeneity and hardness distribution has not been discussed. Therefore, the influence of heat treatment process, the compositions and microstructure distribution on the properties of key area of wheel rim need to be further studied and explored. In this paper, the high throughput quantitative distribution characterization method of composition, microstructure and hardness of high-speed railway wheel materials have been studied. the high throughput scanning electron microscope was used to automatically acquire the microstructure images of a large size area of the rim tread for the first time. Combined with the image batch processing method, the quantitative distribution map of ferrite area fraction on the key area of wheel was obtained. At the same time, the scanning electron microscope (SEM) combined with high performance energy spectrum technology were used and the original position quantitative statistical distribution characterization of the inclusions near the tread area of the wheel rim has been performed. The statistical mapping correlations of composition, microstructure and micro hardness on the same location of wheel rim were investigated, and the effects of wheel rim-chilling process on the ferrite distribution and Vickers hardness distribution were also discussed.

## 2. Materials and Methods

### 2.1. Composition and Process of Experimental Samples

Two samples were cut from the rim region of high-speed railway wheel. One sample was used for the content determination the of Cr, Mo, V, Cu, Al, Mn, Si, P, C, and S by spark discharge atomic emission spectrometric method (Spark-AES) [23] and another sample was used for the determination of oxygen element by pulse heating inert gas fusion-infra-red absorption method. The average content of main elements and the standard deviation of the results for three measurements are shown in Table 1. Composite deoxidation process was adopted with total oxygen content less than 0.002%. Stage quenching method was applied for heat treatment as described in literature [1] and water spray cooling was adopted only for tread area of wheel rim.

### 2.2. Quantitative Distribution Characterization of Compositions and Inclusions in Rim Region

**Figure 1:**
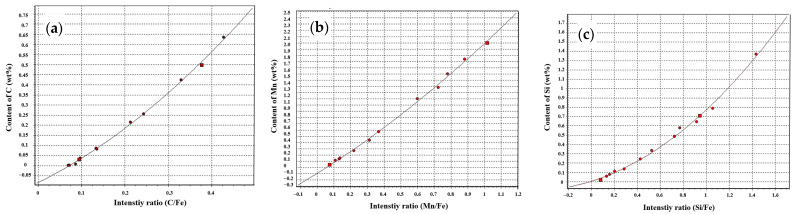
Calibration curve of content for different elements. (**a**) C, (**b**) Mn, and (**c**) Si.

Metal original position analyzer (OPA 200, NCS Testing Technology Co., Ltd., Beijing, China) was used to analyze the elemental distribution on wheel rim. The surface of samples was ground on the resin paper with the size of 46 meshes. The scanning mode is linear at a continuous scanning speed of 1 mm/s along X-axis. Step model is used along Y-axis with an interval of 2 mm. The parameters of OPA are as fol-lows: Exciting frequency: 500 Hz; exciting capacitance: 7.0 μF; exciting resistance:6.0 Ω; spark gap: 2.0 mm; purity of argon: 99.999%; flux of argon: 80 mL/s; material for electrode: tungsten electrode with 45 corner angle and the diameter of 3 mm [24]. Through high-speed data acquisition and analysis of the spectral signals generated by spark discharge and continuous scanning excitation, the content and distribution information of elements at different positions in the sample surface can be obtained. According to the element content of the sample to be tested, a series of micro-alloyed steel standard samples named with gsb-03-2453-2008 were chosen as the quantitative calibration samples to obtain the content calibration curve as shown in Figure 1. It was found that the regression calibration curves of most elements had good linear correlation, and the determination coefficient was about 0.99. Therefore, these calibration curves have been used for quantitative analysis of elemental distribution of wheel samples.

**Figure 2:**
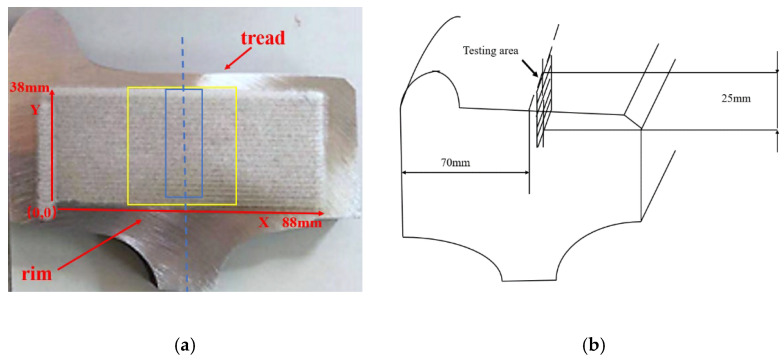
Analytical area of sample: (**a**) for composition, microstructure and hardness analysis; (**b**) for inclusion analysis.

The sample analysis area is shown in Figure 2a, and the size of the analysis area is 88 × 38 mm^2^.

The inclusions along the rolling direction of the wheel have been analyzed. The inclusion sampling and testing area at the wheel rim is shown in Figure 2b. The sample with the size of 15 × 25 × 10 mm^3^ was cut from the wheel rim. After the process of grinding, fine grinding, polishing and absolute ethanol cleaning of the surface, the sample was analyzed by SEM (Vega3, Tescan Co. Czech, Brno, Czech Republic) combined with the particle identification and analysis module of energy dispersive spectrometer (EDS) (Aztec, Oxford, UK). The analysis area was 12 × 20 mm^2^. Aluminum foil was pasted to the edge of the sample to calibrate the gray scale of the electron microscope image. By adjusting the appropriate brightness and contrast and setting the suitable brightness threshold value for inclusion recognition, inclusion particles have been identified. Then the composition of inclusions was determined by EDS analysis automatically.

### 2.3. Quantitative Distribution Characterization of Microstructure and Hardness on the Wheel Rim

The block sample with the size of 38 × 50 mm^2^ along the area marked with the yellow box as shown in Figure 2a was cut from the wheel rim. The polished sample was eroded by alcohol solution containing 3% of nitric acid (volume fraction) with the etching time of 10 s and then the microstructure of the eroded sample surface was examined by high throughput field emission scanning electron microscope (Navigator OPA, NCS Testing Technology Co., Ltd., Beijing, China). The microstructure images from the area presented in Figure 2a marked with the blue box were automatically and continuously acquired and the test area was 30 × 10 mm^2^. With the help of image batch processing module, the quantitative statistical distribution characterization of ferrite area fraction from the acquisition images with the number of 6256 was obtained.

A hardness map of area marked in Figure 2a with the yellow box was conducted by automatic micro hardness tester (Q10 A+, Qness. Co., Salzburg, Austria) after the process of rough grinding, fine grinding, polishing and absolute ethanol cleaning. The test load was 0.5 kg with the loading time of 15 s and the transverse and longitudinal scanning spacing was 1 mm. The test area was 35 × 45 mm^2^.

## 3. Results

### 3.1. Elemental Distribution in Rim Area of Railway Wheel

**Figure 3:**
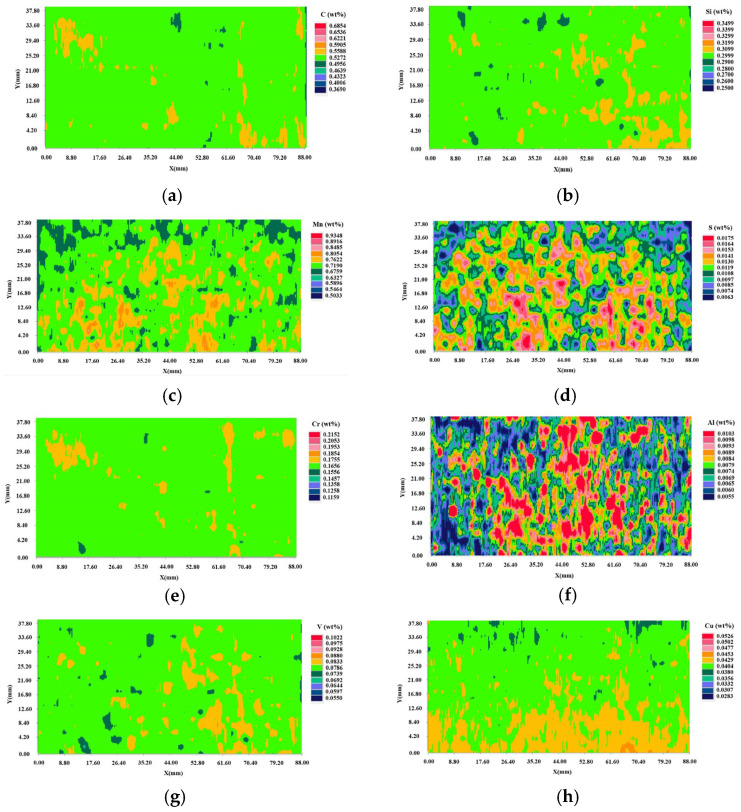
Quantitative distribution results of elements, (**a**–**h**) 2D content distribution of C, Si, Mn, S, Cr, Al, V, and Cu.

The quantitative distribution results of components are shown in Figure 3. The maximum degree of segregation is the ratio of the element content at the location to the average content of the whole tested area. The statistical results are shown in Table 2. Statistical segregation degree of the elements was calculated by the formula as follows:

S = (C_2_ − C_1_)/2C_0_ (1)

where C_0_ is the median value of the content, C_1_ and C_2_ are the upper and lower limits of the 95% content confidence interval respectively. The greater the statistical segregation value, the more serious the segregation is. It was found that there was serious segregation of S and Al elements and many dotted red areas with higher content appeared in the two-dimensional distribution map as shown in Figure 3. Therefore, the statistical segregation degree of S and Al exceeded 0.1 and the values of maximum segregation degree were also larger than other elements. It was found that the segregation trend of S and Mn presented on the two-dimensional distribution map was also very similar as shown in Figure 3e,g. The content of Mn in the wheel increased from 0.705% to 0.815% within the radial depth of 12−34 mm below the tread surface, and the content of S increased by 0.006% from the tread surface to the 30 mm radial depth below the tread. The distribution of C, Si, Cr, and V elements was relatively homogeneous and the values of the statistical segregation degree for these elements were all less than 0.05.

### 3.2. Distribution of Inclusions near Tread Area in Rim Center

**Figure 4:**
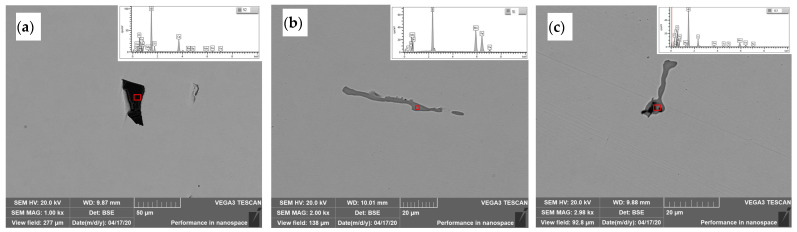
Morphology and composition of different types of inclusions in rim region. (**a**) Oxide; (**b**) sulfide; (**c**) complex inclusion.

**Figure 5:**
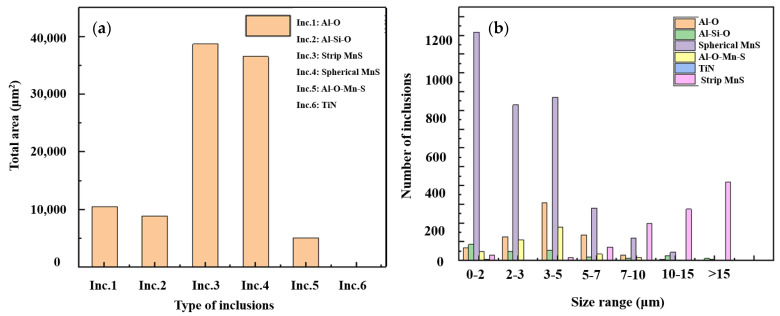
Area fraction and particle size distribution of different types of inclusions. (**a**) Area for different inclusions; (**b**) number of different inclusions.

**Figure 6:**
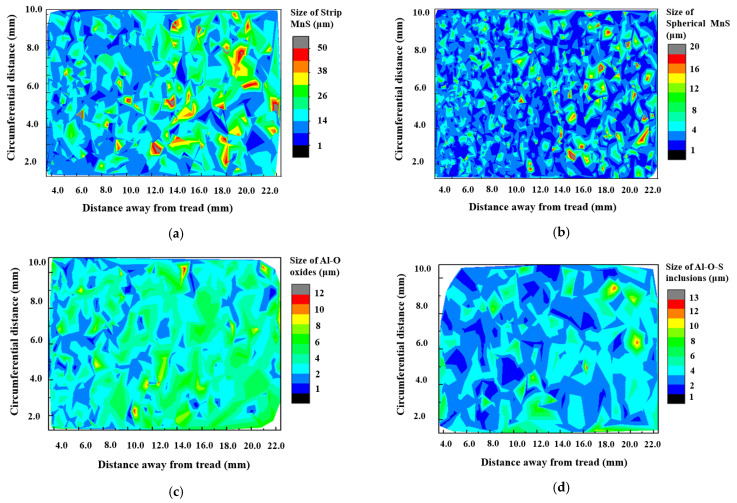
Two-dimensional size distribution of different types of inclusions. (**a**) Elongated sulfides; (**b**) spherical sulfides; (**c**) oxides; (**d**) complex inclusions.

Figure 4 shows the typical inclusion morphologies and the compositions was determined by EDS from of the regions marked with red circle. It indicates that there were four main type of inclusions such as oxides, elongated sulfides, spherical sulfides and oxysulfides (oxide-sulfide complex inclusions) existed in the wheel rim. As a result of the application of inclusion modification process, the oxysulfides mainly appeared in the form of sulfides enveloping oxides as shown in Figure 4c. The oxides enveloped by the sulfides were mostly alumina and spinel inclusions. The number of inclusions was counted by the particle detection module included in the energy spectrum analysis software. A small amount of silicon and titanium inclusions were also found and the area fraction and size distribution of different types of inclusions are shown in Figure 5. The two-dimensional size distribution of different inclusions is shown in Figure 6. The inclusions at the wheel rim were mainly single ellipsoidal or elongated sulfides (MnS), and their area fraction and quantity were much higher than those of oxides. The length of some elongated MnS inclusions was more than 50 μm and the volume fraction was the largest in all types of inclusions of the wheel. Most of the ellipsoidal sulfide particles are less than 5 μm in size. The oxide inclusions were mainly aluminum oxide with irregular polygon shape with the size of 3−5μm and the number of oxides larger than 10 μm was very small. There was no oxides and complex inclusions with size larger than 15 μm found in the rim area. At a depth of 10 to 20 mm below the tread surface, the length of elongated MnS inclusions increased significantly, and the number of large spherical sulphides also increased slightly as shown in Figure 6. The size of oxides and their complex inclusions also increases to a certain extent at the depth of 10 to 20 mm below the tread than that near the tread surface.

### 3.3. Ferrite Structure and Micro Hardness Distribution in the Central Area of Rim

**Figure 7:**
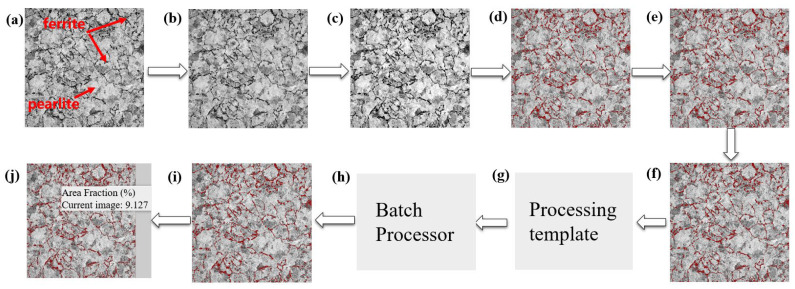
Batch processing flow of wheel microstructure image. (**a**) Original image from SEM; (**b**) image denoising; (**c**) contrast adjustment of the feature image; (**d**) image segmentation through gray threshold; (**e**) feature recognition of microstructure; (**f**) small holes removed from the recognized features; (**g**) processing template of single image generated; (**h**) image batch processing by the generated template; (**i**) manual correction for some images incorrectly identified; (**j**) ferrite area fraction obtained on different region of wheel rim.

**Figure 8:**
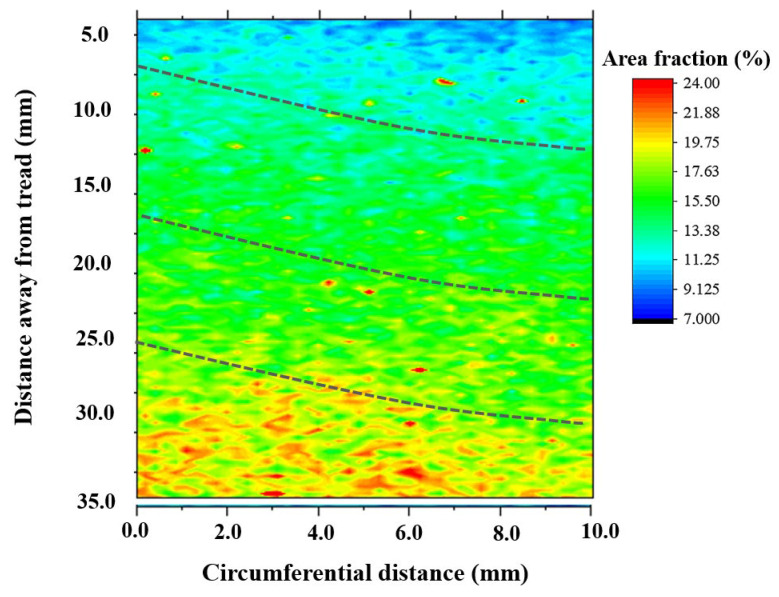
2D distribution of ferrite area mass fraction.

**Figure 9:**
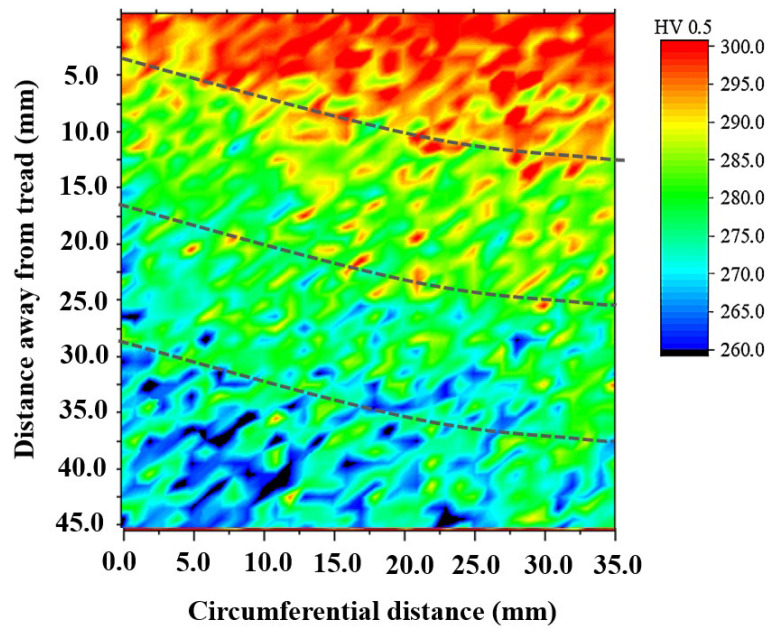
2D distribution of hardness.

The microstructure in the wheel rim was composed of a lamellar pearlite with a small amount pre-eutectoid ferrite (dark region in secondary electron (SE) image of Figure 7) mainly distributed along the prior austenite grain boundary. The quantitative processing method of microstructure images acquired from high throughput SEM was studied. According to the gray difference of the two-phase structure in the wheel, the effective extraction and segmentation of ferrite structure were obtained after noise reduction, contrast adjustment, threshold segmentation, feature stripping and debris removal as shown in Figure 7. The image processing template was generated and was used to process all microstructure images acquired in bulk to realize the segmentation and recognition of ferrite structure. After further confirmation and correction manually, the area fraction of ferrite on different positions of rim was obtained. The two-dimensional distribution of ferrite area fraction in the central area of rim is shown in Figure 8. It was found that area fraction of ferrite was gradually increasing from trend surface to the inside area. In the radial depth of 5 mm below the tread surface, the ferrite area fraction was about 10%, but in the radial depth of 35 mm away from the tread, the ferrite area fraction increased to more than 20%. The distribution of circumferential ferrite was asymmetrical and the ferrite area fraction near the flange was significantly higher than that on the other side. The two-dimensional distribution of micro hardness below the tread surface in the rim center area is shown in Figure 9. The hardness distribution trend was just opposite to the ferrite area fraction distribution. The Vickers hardness value varied in range from 260 HV to 290 HV, with the trend of increasing hardness toward the tread surface and toward the right-hand (field) side of the wheel rim.

Fatigue failure can easily occur at very high number of cycles when the ferrite content is higher. This also indicates that the influence of ferrite on the VHCF behavior becomes greater when the ferrite content is higher. Hui et al. [25] showed that fatigue crack easily initiates at ferrite or ferrite/pearlite boundary and propagates preferentially along that boundary in medium-carbon microalloyed steels with ferrite content of 40−50%. For medium-high caron pearlitic wheels. However, the ferrite content in the rim is relatively lower (generally less than 20%). Thus, it may imply that the ferrite has no considerable effect on the very high cycle fatigue behavior in wheel steels.

The relationship between conventional fatigue limit and Vickers hardness for the wheel steels is consistent with the results for most medium and low strength steels (hardness less than 400 HV) reported by Murakami et al. [26]. When the rotating bending fatigue or uniaxial fatigue behavior of smooth specimens is determined by the micro-structural factor, there is a good linear correlation between fatigue strength and Vickers hardness of steel matrix: σw = (1.6 ± 0.1) HV. So, the fatigue strength can be predicted by the presented equation if the Vickers hardness of steel matrix has been obtained [7].

## 4. Discussion

### 4.1. Influence of Inclusions Distribution on the Element Segregation

**Figure 10:**
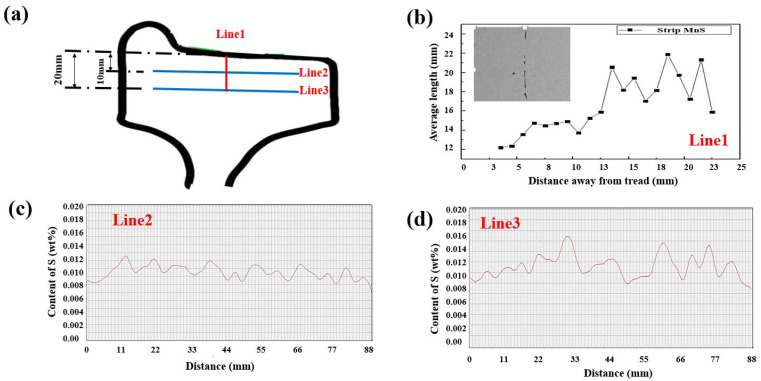
Correlation between strip sulfide distribution and content distribution of S. (**a**) Analytical area; (**b**) variation of average length of sulfides from the depth of 4 mm to 23 mm below the tread surface; (**c**) content distribution of S in the depth of 10 mm below the tread surface; (**d**) content distribution of S in the depth of 20 mm below the tread surface.

The size distribution of long strip sulfides has a great impact on the segregation degree of sulfur elements. From the radial depth of 10 mm to 20 mm below the tread surface, the number of strip sulfides with a length of more than 15 microns has increased significantly which resulted the increase of average length of elongated MnS sulfides as shown in Figure 10b. With the increasing distance below the tread surface, content fluctuation of S element became more and more obvious. In the location of line 2 as shown in Figure 7c, the varied range of S content was mainly between 0.009% and 0.012% with the statistic segregation of 0.142. However, when the radial depth increased to 20 mm as shown in line 2 of Figure 7, the content range of S elements was significantly expanded and the maximum content value exceeded 0.016%, which resulted in a larger segregation degree of S element. In the radical depth of 20 mm below the tread surface, the value of S segregation degree was increased to 0.240, which is much higher than in the radical depth of 10 mm. So, it can be concluded that with the length increase of elongated MnS sulfides, the segregation degree of S element increased.

The tested wheel material had a moderate sulfur content with the average content of 0.011%. Indeed, inclusions behave as stress raisers, due to the elastic-plastic strain incompatibilities with the steel matrix and are preferential sites for damage initiation [27,28]. The size and shape of the inclusions are the main parameters that will influence the very-high-cycle fatigue (VHCF) property. By increasing sulfur content, the fracture toughness of wheel material can be improved by inclusion modification techniques through sulfides (MnS) enveloping oxides, which reduces effectively the stress concentration generated by the oxides [15]. The size of these oxides enveloped by sulfides ranged mainly from 1 to 5 μm and inclusions larger than 10 μm were rarely observed. For VHCF, the fatigue strength is inversely proportional to the inclusion size and the heterogeneous distribution of the inclusion will increase the risk of VHCF. Through the modified deoxidation technology the size of near-globular inclusions of this railway wheel decreased to less than 10 μm so that the formation probability of inclusion cluster is significantly reduced and the VHCF behavior can be improved [29].

### 4.2. The Relationship between Quenching Process, Microstructure and Hardness Distribution of Wheel

**Figure 11:**
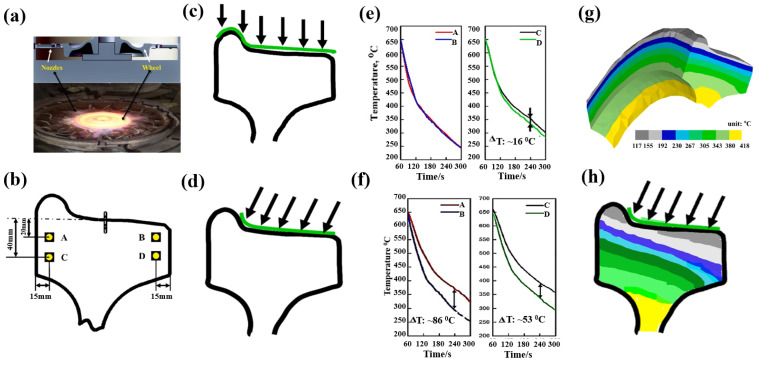
Temperature distribution in different regions of wheel rim under water spray quenching process of tread. (**a**) Wheel quenching process; (**b**) location of temperature measuring points; (**c**) spraying water process at a vertical angle to tread; (**d**) spraying water process at an oblique angle to tread; (**e**) cooling curve at a vertical angle; (**f**) cooling curve at an oblique angle; (**g**) calculated temperature field distribution at a vertical angle; (**h**) determined temperature distribution at an oblique angle.

**Figure 12:**
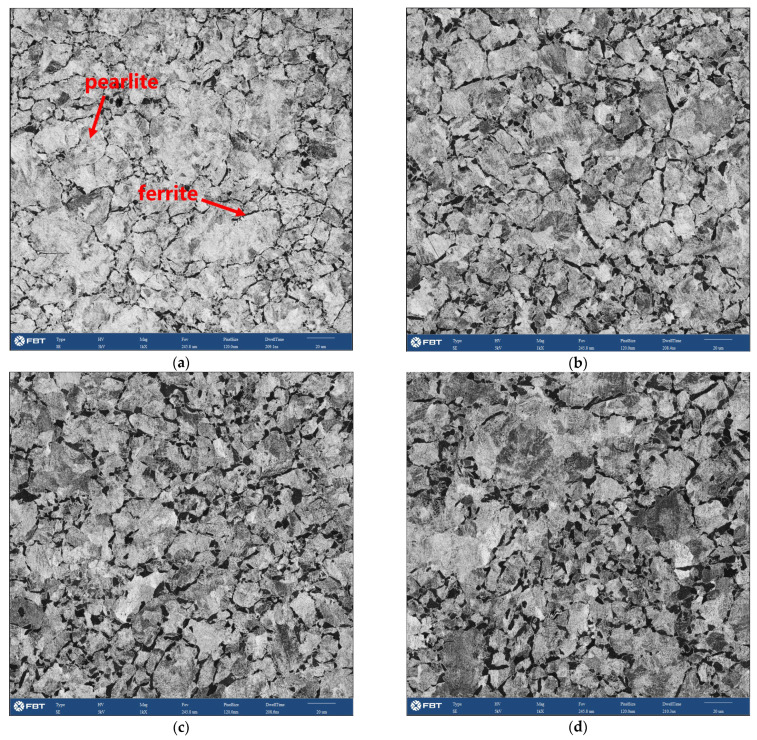
Microstructure morphology at different positions from tread, (**a**) 5 mm, (**b**) 15 mm, (**c**) 25 mm, and (**d**) 35 mm.

**Figure 13:**
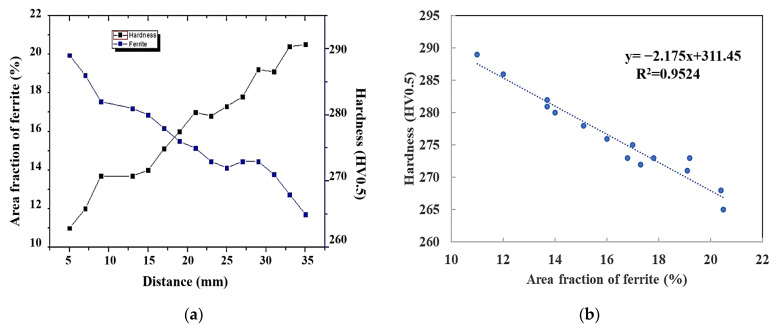
Mapping correlation between ferrite area fraction and micro Vickers hardness. (**a**) Distribution in different depth away from tread for area fraction of ferrite and vickers hardness; (**b**) Hardness variation with the change of area fraction of ferrite.

The wheel cooling process was carried out by stage quenching [1]. The tread was quenched by water spraying. It was found that there were gradient distributions of different temperature fields from the tread to the inside area (Figure 11). The closer the tread area was, the faster the cooling rate was, which resulted in the much lower temperature of the near tread than that of the area near wheel spoke. However, different spray angles would also lead to differences in temperature field distribution. When the angle of water flow was just perpendicular to the tread, as shown in Figure 11c, the temperatures of the upper and lower surfaces of the wheel were basically similar, so the temperature of point A and B was basically similar, and the temperature of points C and D was not different, as shown in Figure 11e. However, the water flow angle cannot be completely perpendicular to the tread, and there will be an inclined angle, as shown in Figure 11d. Therefore, the water flow away from the wheel flange will contact the tread for a longer time. The cooling rate in this area was faster than that at the rim end, resulting in the inconsistency of the temperature drop curve between the wheel flange and the other side, as shown in Figure 11f. Therefore, the temperature field distribution after 300 s of continuous water spraying was also quite different from that of vertical tread water spraying, as shown in Figure 11d,h. The unsynchronized cooling on both sides of the rim alters the phase behavior of ferrite and pearlite. The ferrite grain size after continuous cooling transformation becomes finer as austenite grain size is refined. Therefor, the ferrite near the tread was fine, and presents a network distribution along the original grains with lower area fraction [30]. The ferrite near the tread was fine, and presents a network distribution along the original grains. The grains were also relatively uniform and fine, so the ferrite area fraction was low. But the area far away from the tread stays at a higher temperature for a long time, resulting in the coarseness of the ferrite structure, as shown in Figure 12. The distribution trend of ferrite area fraction and micro vickers hardness along the line 1 of Figure 8 from 5 mm to 35 mm away from the tread is shown in Figure 13a. It can be seen that the linear distribution trend of ferrite structure area fraction and vickers hardness was completely opposite. Based the quantitative characterization of area fraction and micro hardness on the same location of wheel rim, a statistical mapping relationship between ferrite area fraction and Vickers hardness was investigated. Through binary linear fitting of the two groups of parameters, it can be found that there is a good negative correlation between ferrite iron area fraction and micro vickers hardness, and its linear correlation coefficient exceeds 0.97, It shows that the ferrite structure near the tread has an important influence on the wheel hardness. Moreover, the rapid quenching made the wheel tread area harder than the wheel rim centre and thus more resistant to wear and crack initiation.

## 5. Conclusions

In this paper, high throughput quantitative distribution characterization methods for the composition, microstructure, inclusions, and micro hardness of high-speed wheel materials have been studied. The quantitative distribution analysis of ten elements in the rim area was carried out. The distribution trend of the content of nine elements, size and quantity of sulfides and oxides, ferrite area fraction and Vickers hardness from the wheel tread surface to the radial depth of about 50 mm has been discussed. The following conclusions are obtained:

(1) There was serious segregation of S and Al elements on the region of wheel rim with the statistical segregation degree of S and Al exceeded 0.1. The segregation trend of S and Mn presented on the two-dimensional distribution map was also very similar. The distribution of C, Si, Cr, and V elements was relatively homogeneous and the values of the statistical segregation degree for these elements were all less than 0.05.
(2) The size distribution of long strip sulfides has a great impact on the segregation degree of sulfur elements. With the length increase of elongated MnS sulfides, the segregation degree of S element increased. Inclusion modification techniques through sulfides (MnS) enveloping reduced effectively the stress concentration generated by the oxides and the size of these oxides enveloped by sulfides ranged mainly from 1 to 5 μm and inclusions larger than 10 μm were rarely observed.
(3) Asymmetrical graded distribution of ferrite area fraction and micro hardness appeared from the tread surface to the radical depth of 35−50 mm below the surface as a result of the unsynchronized cooling on both sides of the rim. Due to the influence of tread water spray cooling process, the phase behavior of ferrite was altered. In the radial depth of 5 mm below the tread surface, the ferrite area fraction was about 10%, but in the radial depth of 35 mm away from the tread, the ferrite area fraction in-creased to more than 20%. The distribution of ferrite structure at the rim has a great impact on the hardness distribution. The micro hardness reduced linearly with radical depth below the tread surface the increase of ferrite area fraction. The Vickers hardness value varied in range from 260 HV to 290 HV, with the trend of increasing hardness toward the tread sur-face and toward the right-hand (field) side of the wheel rim.

## Figures and Tables

**Table 1:** The content of main elements in high-speed railway wheel by Spark-AES (wt %).

Sample	C	Si	Mn	P	S	Cr	Cu	Mo	V	Al	Fe
D2	0.520	0.304	0.720	0.006	0.011	0.167	0.039	0.008	0.087	0.009	98.129
SD	0.0041	0.0022	0.0044	0.0005	0.0010	0.0019	0.0035	0.0009	0.0015	0.0008	0.0208

**Table 2:** Statistical distribution analysis results of each element.

Element	Average Content%	Maximum Segregation Degree	Position (X, Y) *	Statistical Segregation Degree
C	0.527	1.072	(22.59, 4.05)	0.0283
Si	0.305	1.032	(36.37, 5.95)	0.0166
Mn	0.728	1.133	(28.16, 4.05)	0.0556
S	0.010	1.527	(59.84, 10.01)	0.2926
Cr	0.166	1.067	(80.67, 25.97)	0.0247
Cu	0.040	1.080	(45.76, 5.95)	0.0409
V	0.084	1.083	(28.16, 4.05)	0.0316
Al	0.008	4.189	(54.56, 25.97)	0.3900

* Location of maximum segregation.

## Data Availability

All data included in this study are available upon request by contact with the corresponding author.

## References

[1] Paul M.B., Claire D., Adam B. (2014). The Influence of Wheel/Rail Contact Conditions on the Microstructure and Hardness of Railway Wheels. Sci. World J..

[2] Liu C.P., Zhao X.J., Liu P.T., Pan J.Z., Ren R.M. (2019). Influence of Contact Stress on Surface Microstructure and Wear Property of D2/U71Mn Wheel-Rail Material. Materials.

[3] Liu C.-P., Pan J.-Z., Liu P.-T., Ren R.-M., Zhao X.-J. (2020). Influence of original microstructure on rolling contact fatigue property of D2 wheel steel. Wear.

[4] Chen Y.D., Ren R.M., Zhao X.J., Chen C.H., Pan R. (2020). Study on the surface microstructure evolution and wear property of bainitic rail steel under dry sliding wear. Wear.

[5] Zhou S.T., Li Z.D., Jiang L., Wang X., Xu P., Ma Y.X., Yan Y.P., Yang C.F., Yong Q.L. (2022). An investigation into the role of non-metallic inclusions in cleavage fracture of medium carbon pearlitic steels for high-speed railway wheel. Eng. Fail. Anal..

[6] Gong T., Liu X.L., Wu S., Zhang G.Z., Chen E.Q., Qian G.A., Berto F. (2021). Study on damage tolerance and remain fatigue life of shattered rim of railway wheels. Eng. Fail. Anal..

[7] Li Z.D., Zhou S.T., Yang C.F., Yong Q.L. (2019). High/very high cycle fatigue behaviors of medium carbon pearlitic wheel steels and the effects of microstructure and non-metallic inclusions. Mater. Sci. Eng. A.

[8] Zhang G.Z., Ren R.M. (2019). Study on typical failure forms and causes of high-speed railway wheels. Eng. Fail. Anal..

[9] Diao G., Yan Q., Shi X., Zhang X., Wen Z., Jin X. (2019). Improvement of wear resistance in ferrite-pearlite railway wheel steel via ferrite strengthening and cementite spheroidization. Mater. Res. Express.

[10] Rezende A.B., Fonseca S.T., Miranda R.S., Fernanes F.M., Grijalba F.A.F., Farina P.F.S., Mei P.R. (2021). Effect of niobium and molybdenum addition on the wear resistance and the rolling contact fatigue of railway wheels. Wear.

[11] Zuo Y., Zhou S.T., Li Z.D., Pan T., Xiang J.Z., Yong Q.L. (2016). Effect of V and Si on Microstructure and Mechanical Properties of Medium-carbon Pearlitic Steels for Wheel. Chin. J. Mater. Res..

[12] Shi J.P., Liu Z.X., Xiao Q. (2012). The effect of chemical composition, microstructure on the mechanical properties of wheel steels. China Railw. Sci..

[13] Zeng D.F., Lu L.T., Gong Y.H., Zhang N., Gong Y.B. (2016). Optimization of strength and toughness of railway wheel steel by alloy design. Mater. Des..

[14] Ma Y., Pan T., Jiang B., Cui Y.H., Su H. (2011). Study of the effect of sulfur contents on fracture toughness of railway wheel steels for high speed train. Acta Metall. Sin..

[15] Sourmail T., Garcia C.M., Caballero F.G., Cazottes S., Epicier T., Danoix F., Milbourn D. (2017). The Influence of Vanadium on Ferrite and Bainite Formation in a Medium Carbon Steel. Metall. Mater. Trans. A.

[16] Gao B., Tan Z.L., Liu Z.N., Gao G.H., Zhang M., Zhang G.Z. (2019). Influence of non-uniform microstructure on rolling contact fatigue behavior of high-speed wheel steels. Eng. Fail. Anal..

[17] Zhang R.J., Zheng C.L., Lv B., Zhang P.J., Gao G.H., Yang Y.Q., Zhang F.C. (2022). Effect of non-uniform microstructure on rolling contact fatigue performance of bainitic rail steel. Int. J. Fatigue.

[18] Li G., Hong Z.Y., Yan Q.Z. (2015). The influence of microstructure on the rolling contact fatigue of steel for high-speed-train wheel. Wear.

[19] Liu X.D., Liu P.T., Zhao X.J., Ren R.M. (2021). Influence of Original Microstructure on Rolling Contact Fatigue Properties of ER9 Wheel Steel. Thibology.

[20] Zhang X.F., Jia Y.H., Sheng L., Yuan L.J., Li J.Q. (2022). Characterization of segregation degree for large size metal component and application on high-speed train wheel. Anal. Chim. Acta.

[21] Suetrong C., Uthaisangsuk V. (2022). Investigation of fatigue crack propagation in ER8 railway wheel steel with varying microstructures. Mater. Sci. Eng. A.

[22] Tian Y., Tan Z.L., Wang J., Wang R., Liu Y.R., Zhang M. (2022). Experiment and finite element analysis of asymmetrical hardness induced by quenching in railway wheel. Eng. Fail. Anal..

[23] Jia Y.H., Cheng H.M., Yang J.W., Sun J.J. (2017). Key points and application of revised spark discharge atomic emission spectrometry standard including GB/T 14203-2016 and GB/T 4336-2016. Metall. Anal..

[24] Li D.L., Wang H.Z. (2014). Original position statistic distribution analysis for the sulfides in gear steels. ISIJ Int..

[25] Hui W., Chen S., Zhang Y., Shao C., Dong H. (2015). Effect of vanadium on the high-cycle fatigue fracture properties of medium-carbon microalloyed steel for fracture splitting connecting rod. Mater. Des..

[26] Murakami Y., Kodama S., Konuma S. (1989). Quantitative evaluation of effects of non-metallic inclusions on fatigue strength of high strength steels. I: Basic fatigue mechanism and evaluation of correlation between the fatigue fracture stress and the size and location of non-metallic inclusions. Int. J. Fatigue.

[27] Stiénon A., Fazekas A., Buffière J.-Y., Vincent A., Daguier P., Merchi F. (2009). A new methodology based on X-ray micro-tomography to estimate stress concentrations around inclusions in high strength steels. Mater. Sci. Eng. A.

[28] Pan T., Yang Z.G., Bai B.Z., Fang H.S. (2003). Study of thermal stress and strain energy in γ-Fe matrix around inclusion caused by thermal coefficient difference. Acta Metall. Sin..

[29] Cong T., Qian G.A., Zhang G.Z., Wu S., Pan X.N., Du L.M., Liu X.L. (2021). Effects of inclusion size and stress ratio on the very-high-cycle fatigue behavior of pearlitic steel. Int. J. Fatigue.

[30] Furuuhara T., Kikumoto K., Saito H., Sekine T., Ogawa T., Morito S., Maki T. (2008). Phase transformation from fine grained austenite. ISIJ Int..

